# Poor Metabolizers at the Cytochrome P450 2C19 Loci Is at Increased Risk of Developing Cancer in Asian Populations

**DOI:** 10.1371/journal.pone.0073126

**Published:** 2013-08-27

**Authors:** Hong Wang, Kang Song, Zenggan Chen, Yanmin Yu

**Affiliations:** 1 Department of General Surgery, Zhongshan Hospital, Fudan University, Shanghai, People’s Republic of China; 2 Liver Cancer Institute, Zhongshan Hospital, Fudan University, Shanghai, People’s Republic of China; 3 Department of Orthopedics, Zhongshan Hospital, Fudan University, Shanghai, People’s Republic of China; 4 Department of Breast Surgery, Huangpu Central Hospital of Shanghai, Shanghai, People’s Republic of China; Baylor College of Medicine, United States of America

## Abstract

**Background:**

CYP2C19 encodes a member of the cytochrome P450 superfamily of enzymes, which play a central role in activating and detoxifying many carcinogens and endogenous compounds thought to be involved in the development of cancer. In the past decade, two common polymorphisms among CYP2C19 (CYP2C19*2 and CYP2C19*3) that are responsible for the poor metabolizers (PMs) phenotype in humans and cancer susceptibility have been investigated extensively; however, these studies have yielded contradictory results.

**Methods and Results:**

To investigate this inconsistency, we conducted a comprehensive meta-analysis of 11,554 cases and 16,592 controls from 30 case-control studies. Overall, the odds ratio (OR) of cancer was 1.52 [95% confidence interval (CI): 1.23–1.88, *P*<10^-4^] for CYP2C19 PMs genotypes. However, this significant association vanished when the analyses were restricted to 5 larger studies (no. of cases ≥ 500 cases). In the subgroup analysis for different cancer types, PMs genotypes had an effect of increasing the risks of esophagus cancer, gastric cancer, lung cancer and hepatocellular carcinoma as well as head neck cancer. Significant results were found in Asian populations when stratiﬁed by ethnicity; whereas no significant associations were found among Caucasians. Stratiﬁed analyses according to source of controls, significant associations were found only in hospital base controls.

**Conclusions:**

Our meta-analysis suggests that the CYP2C19 PMs genotypes most likely contributes to cancer susceptibility, particularly in the Asian populations.

## Introduction

Cancer is considered to be a multifactorial disease, in which multiple exposures to endogenous factors and dietary carcinogens interact with individual genetic background in a complex manner resulting in modulation of the risk. It has been reported that up to 80% of human cancers arise as a consequence of environmental exposure and host susceptibility factors [1]. Most pro-carcinogens exert their genotoxicity after undergoing metabolic activation by various enzymes [2]. Therefore, polymorphisms of the genes encoding for those enzymes involved in the activating and detoxifying carcinogens and endogenous compounds may be related to inter-individual differences in cancer susceptibility.

Individuals vary widely in their susceptibility to carcinogens. One attractive genetic mechanism to account for this variability is the activity of polymorphically expressed cytochrome P450 enzymes that activate procarcinogens or conversely detoxify carcinogens. Cytochrome P450 2C19 (CYP2C19) is an enzyme involved in the metabolism of an extensive range of clinical agents including diazepam, mephenytoin, proton-pump inhibitors and clopidogrel [3]. CYP2C19 also plays a crucial role in either the detoxiﬁcation or inactivation of potential carcinogens, or the bioactivation of some environmental procarcinogens to reactive DNA-binding metabolites, such as nitrosamine [4]. Poor metabolizer (PM) and extensive metabolizer (EM) phenotypes has been demonstrated based on the ability to metabolize (S)-mephenytoin and other CYP2C19 substrates which have been assigned to genetic polymorphisms [5,6]. Several important single nucleotide polymorphisms have been identiﬁed in the CYP2C19 gene; however, most cases can be explained by only two kinds, CYP2C19*2 (rs4244285) and CYP2C19*3 (rs4986893) which are responsible for the PMs phenotype in humans, while EM is assigned to the CYP2C19*1 allele [5,6]. CYP2C19*2 shows a single-base mutation (G→A) in exon 5 of CYP2C19 which produces an aberrant splice site and is known to be present in both Japanese and Caucasian populations. CYP2C19*3 consists of a premature stop codon (G→A) in exon 4 and is reported in Oriental populations including both Japanese and Chinese but rare in the Caucasians [7].

Despite the biological plausibility of CYP2C19 region polymorphisms as a modulator of cancer susceptibility, previously inconsistent results have appeared in the literature. Such inconsistency could be due to the small effect of the polymorphism on cancer, sample size and ethnic diversity, and individual studies may have insufﬁcient power to reach a comprehensive and reliable conclusion. We therefore performed a meta-analysis of the published studies to clarify this inconsistency and to establish a comprehensive picture of the relationship between CYP2C19 and cancer susceptibility.

## Materials and Methods

### Identification of eligible studies

A comprehensive literature search was performed using the PubMed, Web of Science, EMBASE and CNKI (Chinese National Knowledge Infrastructure) databases for relevant articles with a combination of the following keywords: ‘Cytochrome P450 2C19’, ‘CYP2C19’, ‘polymorphism’, ‘variation’, and ‘cancer’, or ‘tumor’, or ‘carcinoma’. Genetic association studies published before the end of December 2012 on cancer and polymorphisms in the CYP2C19 gene described above were retrieved, and their references were checked to identify other relevant publications. Review articles were also inspected to ﬁnd additional eligible studies. All relevant reports identified were included without language restriction. As studies with the same population by different investigators or overlapping data by the same authors were found, the most recent or complete articles with the largest numbers of subjects were included.

### Inclusion criteria and data extraction

The following criteria were used for the study selection: (1) evaluation of at least one of these two polymorphisms (CYP2C19*2 and CYP2C19*3) and cancer risks, (2) original papers containing independent data, (3) identiﬁcation of cancer was conﬁrmed pathologically or histologically, (4) genotype distribution information in cases and controls or odds ratio (OR) with its 95% conﬁdence interval (CI) and P-value and (5) case–control or cohort studies. The major reasons for exclusion of studies were (1) overlapping data, (2) case-only studies and (3) family-based studies.

For each included study, the following information was extracted independently by two investigators (Checklist S1): ﬁrst author’s surname, publication year, ethnicity, cancer type, number of cases and controls, genotyping method, Hardy-Weinberg equilibrium (HWE) status, source of control groups (population-based controls and hospital-based controls), and genotype frequency in cases and controls. For studies including subjects of different ethnic groups, data were extracted separately and categorized as Asians (e.g. Chinese, Japanese), and Caucasians (i.e. people of European origin). Meanwhile, studies investigating more than one kind of cancer were counted as individual data set only in subgroup analyses by cancer type. Cases that were homozygous for either the CYP2C19*2 or CYP2C19*3 mutation (*2/*2 or *3/*3) and heterozygous for CYP2C19*2 and CYP2C19*3 (*2/*3) were categorized as PMs. Cases that were homozygous for the WT (*1/*1) or heterozygous for the WT and mutation (*1/*2 or *1/*3) were categorized as extensive metabolizers (EMs) [3]. The results were compared and disagreements were discussed and resolved with consensus among all authors. Where essential information was not presented in articles, every effort was made to contact the authors.

### Statistical methods

Crude ORs with 95% CIs were used to assess the strength of the association between the CYP2C19 polymorphism and cancer risks. For the CYP2C19 polymorphisms, we estimated the risks of the PMs genotypes on cancer compared with EMs genotypes under recessive model. HWE in the control group was assessed using Fisher’s exact test. Heterogeneity across individual studies was calculated using the Cochran chi-square Q test and I^2^ followed by subsidiary analysis or by random-effects regression models with restricted maximum likelihood estimation. Random-effects and ﬁxed-effect summary measures were calculated as inverse variance-weighted average of the log OR. The results of random-effects summary were reported in the text because it takes into account the variation between studies [8]. In addition, ethnicity, cancer type, source of controls, and sample size were analyzed as covariates in meta-regression. The significance of the overall OR was determined by the Z-test. Funnel plots and Egger’s linear regression test were used to assess evidence for potential publication bias. In order to assess the stability of the result, sensitivity analyses were performed, each study in turn was removed from the total, and the remaining were reanalyzed. All Statistical analyses were done with Stata software version 10.0 (Stata Corporation, College Station, TX, USA). To assess the credibility of genetic associations, the Baysian false discovery probability (BFDP) was calculated [9]. We chose to calculate BFDP values for two levels of prior probabilities: at a medium or low prior level (0.05 to 10^-3^) that would be close to what would be expected for a candidate gene; and at a very low prior level (10^-4^ to 10^-6^) that would be close to what would be expected for a random SNP. The BFDP thresholds of noteworthiness are 0.80 [9]. P values are two-sided at the P = 0.05 level.

## Results

### Characteristics of studies

**Figure 1:**
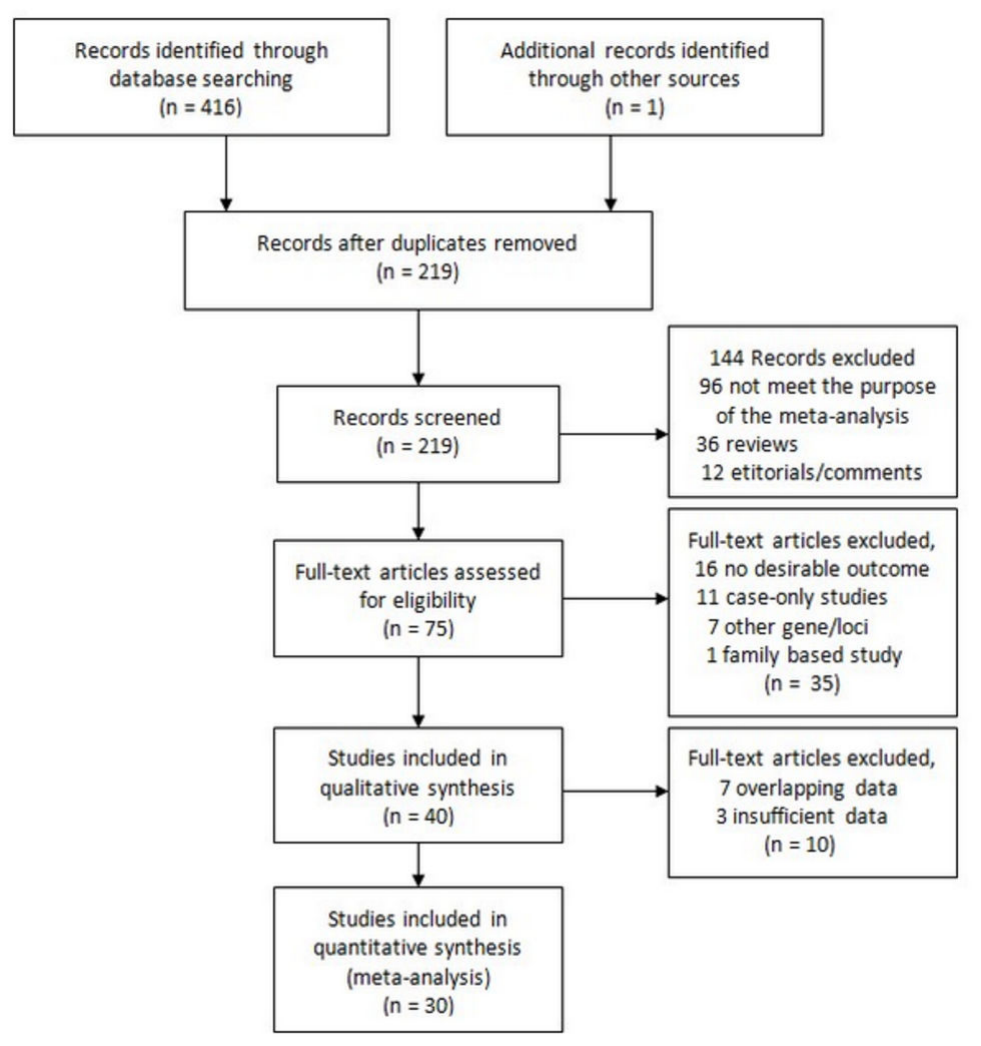
Flow diagram of the study selection process.

The combined search yielded 416 references. 387 articles were excluded because they clearly did not meet the criteria or overlapping references (Figure 1). Finally, a total of 30 studies with 11,554 cancer cases and 16,592 controls examining the association between the CYP2C19 polymorphism and cancer risk were included in the current meta-analysis [3,4,7,10–35]. The polymorphisms were found to occur in frequencies consistent with HWE in the control populations of the vast majority of the published studies. Of the cases, 74.4% were Caucasian, and 25.6% were Asian populations. The detailed characteristics of the studies included in this meta-analysis are presented in Table 1.

**Table 1:** Characteristics of the studies included in the meta-analysis.

Study	Year	Nationality	Ethnicity	Cancer types	No. of case/control	Source of control	Genotyping method
Brockmöller [10]	1996	German	Caucasian	BLC	355/340	Hospital	PCR-RFLP
Tsuneoka [11]	1996	Japanese	Asian	HCC, LC	30/64	Population	PCR-RFLP
Wadelius [12]	1999	Swedish, Danish	Caucasian	PC	178/160	Population	PCR-RFLP
Chau [13]	2000	Japanese	Asian	HCC	29/186	Population	PCR-RFLP
Roddam [14]	2000	British	Caucasian	Leukaemia	557/952	Population	Taqman
Sachse [15]	2002	British	Caucasian	CRC	490/592	Population	PCR-RFLP
Shi [16]	2004	Chinese	Asian	EC, GC, LC BLC	607/372	Population	PCR-RFLP
Mochizuki [17]	2005	Japanese	Asian	HCC	44/843	Population	PCR-RFLP
Sugimoto [18]	2005	Japanese	Asian	GC	111/315	Hospital	PCR-RFLP
Landi [19]	2005	Spanish	Caucasian	CRC	351/321	Population	APEX
Zhou [20]	2006	Chinese	Asian	EC	127/254	Hospital	PCR-RFLP
Tamer [21]	2006	Turkish	Caucasian	CRC, GC	182/105	Hospital	RT-PCR
Xing [22]	2006	Chinese	Asian	BLC	108/112	Hospital	PCR-RFLP
Gemignani [23]	2007	European	Caucasian	LC	245/275	Hospital	Microarray
Jiang [24]	2008	Chinese	Asian	HCC	48/88	Hospital	PCR-RFLP
Yang [25]	2008	Chinese	Asian	CRC	83/112	Hospital	PCR-RFLP
Gra [26]	2008	Russian	Caucasian	Leukemia	83/177	Population	Microarray
Yadav [7]	2008	Indian	Asian	HNC	300/300	Hospital	PCR-RFLP
Khedhaier [27]	2008	Tunisian	Caucasian	BRC	304/240	Population	PCR-RFLP
Sameer [28]	2009	Palestinian	Caucasian	Leukaemia	52/200	Population	PCR-RFLP
Justenhoven [29]	2009	German	Caucasian	BC	969/991	Population	MassARRAY
Zhang [30]	2009	Chinese	Asian	EC	46/38	Hospital	PCR-RFLP
Wen [31]	2009	Chinese	Asian	BLC	87/298	Hospital	Taqman
Isomura [3]	2010	Japanese	Asian	BTC	65/566	Hospital	PCR-RFLP
Chang-Claude [4]	2010	German	Caucasian	BC	3131/5478	Population	MassARRAY
Chang [32]	2010	Chinese	Asian	HCC	68/254	Population	Allele-specific PCR
Gan [33]	2011	Chinese	Asian	BC	600/600	Population	PCR-RFLP
Sainz [34]	2011	German	Caucasian	CRC	1759/1776	Population	SNPlex
Feng [35]	2011	Chinese	Asian	HNC	300/300	Hospital	PCR-RFLP
Unpublished data	/	Chinese	Asian	HCC, CRC	245/283	Hospital	PCR-RFLP

CRC: colorectal cancer; LC: lung cancer; BC: breast cancer; BLC: bladder cancer; EC: esophagus cancer; HCC: hepatocellular carcinoma; GC: gastric cancer; PC: prostate cancer; BTC; biliary tract cancer; HNC: head neck cancer

### Quantitative Data Synthesis

**Figure 2:**
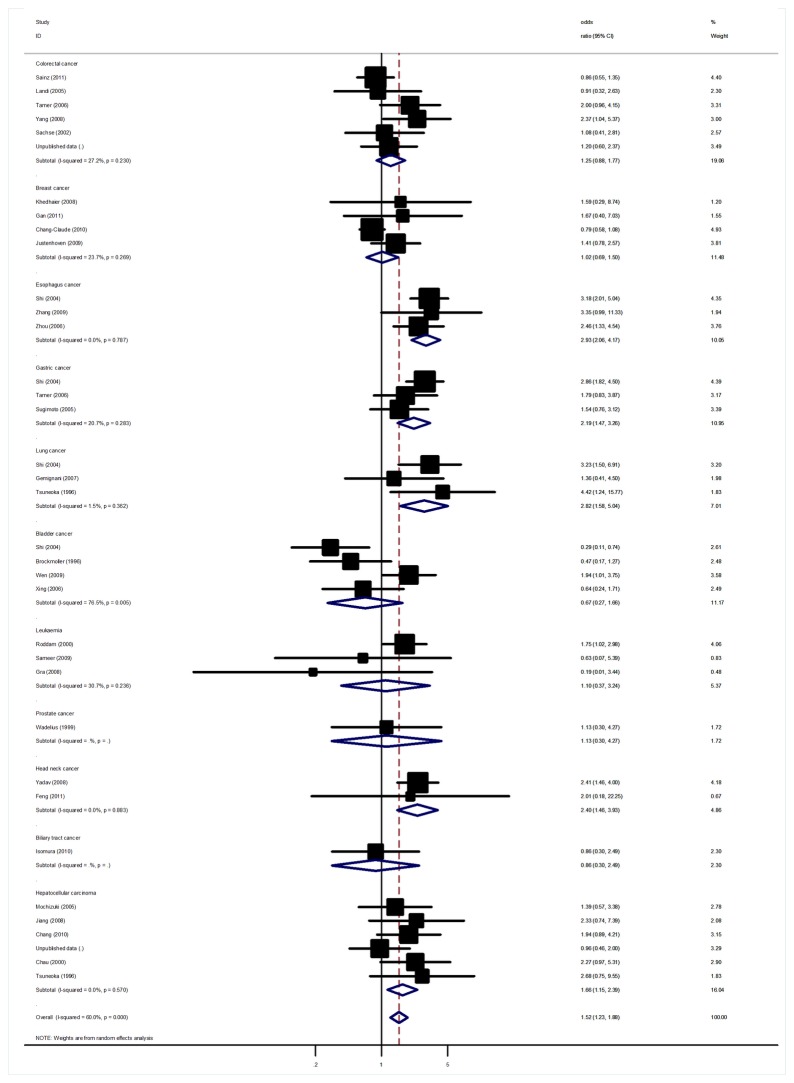
Forest plot from the meta-analysis of CYP2C19 PMs genotypes and cancer risk.

Signiﬁcant heterogeneity was present among the 36 data sets from 30 studies (P<10^-5^). In meta-regression analysis, source of controls (P = 0.13), and HWE status among controls (P = 0.55) did not signiﬁcantly explain such heterogeneity. By contrast, cancer type (P = 0.02), ethnicity (P = 0.006) and sample size (P = 0.01) were signiﬁcantly correlated with the magnitude of the genetic effect, explaining 11%, 17% and 15% of the heterogeneity, respectively. Overall, signiﬁcant associations were found between CYP2C19 PMs genotypes cancer risk when all studies pooled into the meta-analysis. Using random effect model, the summary OR of PMs for cancer was 1.52 [95% CI: 1.23-1.88, P(Z) <10^-4^, P(Q) <10^-5^; Figure 2].

In the stratiﬁed analysis by cancer type, we found that PMs with the minor variant genotypes had a higher risk of esophagus cancer (OR=2.93, 95%CI: 2.06-4.17, P<10^-5^), gastric cancer (OR=2.19, 95%CI: 1.47-3.26, P<10^-4^) and hepatocellular carcinoma (OR=1.66, 95%CI: 1.15-2.39, P=0.006). Furthermore, marginally significant association was also observed for lung cancer and head neck cancer with OR of 2.38 (95%CI: 1.06-5.36, P=0.03) and of 2.40 (95%CI: 1.46-3.93, P=0.001), respectively. However, no signiﬁcant association was found for other types of cancer (Table 2). This analysis is based on pooling of data from a number of different ethnic populations. When stratifying for ethnicity, an OR of 1.84 (95% CI: 1.44-2.35, P <10^-4^) and 1.11 (95% CI: 0.87-1.42, P =0.40) resulted for PMs genotype, among Asian and Caucasian populations, respectively. By considering control source subgroups, the OR was 1.38 (95% CI: 0.96-1.98, P =0.08) in population-based controls compared to 1.56 (95% CI: 1.22-1.98, P <10^-4^) in hospital controls. Subsidiary analyses of HWE status yielded an OR for controls consistent to HWE of 1.42 (95% CI: 1.11-1.80), while similar result was also found for controls deviated from HWE. Analysis restricted to the 5 studies with at least 500 cases, which should be less prone to selective publication than smaller studies, yielded an OR of 1.10 (95% CI: 0.78-1.57, P =0.58) without significant between-study heterogeneity. After applying the BFDP, the polymorphism was identified as a credible positive association (Table S1).

**Table 2:** Results of meta-analysis and subgroup analysis.

Sub-group analysis	No. of data sets	No. cases/controls	OR (95%CI)	P(Z)	P(Q)^a^	I^2^	P(Q)^b^
Overall	36	11554/16592	1.52 (1.23-1.88)	<10^-4^	<10^-5^	60.0%	
Cancer type							<10^-4^
Colorectal cancer	6	2913/3189	1.25 (0.88-1.77)	0.21	0.23	27.2%	
Breast cancer	4	5004/7309	1.02 (0.69-1.50)	0.92	0.27	23.7%	
Esophagus cancer	3	308/664	2.93 (2.06-4.17)	<10^-5^	0.79	0%	
Hepatocellular carcinoma	6	325/1718	1.66 (1.15-2.39)	0.006	0.57	0%	
Gastric cancer	3	336/792	2.19 (1.47-3.26)	<10^-4^	0.28	20.7%	
Lung cancer	3	471/711	2.82 (1.58-5.04)	<10^-4^	0.36	1.5%	
Leukemia	3	692/1329	1.10 (0.37-3.24)	0.86	0.24	30.7%	
Head neck cancer	2	600/600	2.40 (1.46-3.93)	0.001	0.88	0%	
Bladder cancer	4	662/1122	0.67 (0.27-1.66)	0.39	0.005	76.5%	
Prostate cancer	1	178/160	1.13 (0.30-4.27)	0.86	NA	NA	
Biliary tract cancer	1	65/566	0.86 (0.30-2.49)	0.78	NA	NA	
Ethnicity							<10^-4^
Caucasian	14	8656/11607	1.11 (0.87-1.42)	0.40	0.17	26.9%	
Asian	22	2898/4975	1.84 (1.44-2.35)	<10^-4^	0.002	50.6%	
Control source							0.35
Population	19	8901/12885	1.38 (0.96-1.98)	0.08	<10^-4^	72.0%	
Hospital	17	2653/3707	1.56 (1.22-1.98)	<10^-4^	0.14	28.0%	
Sample size							0.0001
No. cases <500	31	4538/6795	1.64 (1.32-2.05)	<10^-5^	0.002	53.4%	
No. cases ≥500	5	7016/9797	1.10 (0.78-1.57)	0.58	0.07	48.1%	
HWE status for controls							0.37
Yes	31	9999/14502	1.49 (1.19-1.88)	0.001	<10^-4^	64.6%	
No	5	1555/2090	1.82 (1.17-2.85)	0.008	0.75	0%	

NA: not available P(Z): Z test used to determine the signiﬁcance of the overall OR.

^a^Cochran’s chi-square Q statistic test used to assess the heterogeneity in subgroups.

^b^Cochran’s chi-square Q statistic test used to assess the heterogeneity between subgroups.

### Sensitivity analyses and Publication bias

**Figure 3:**
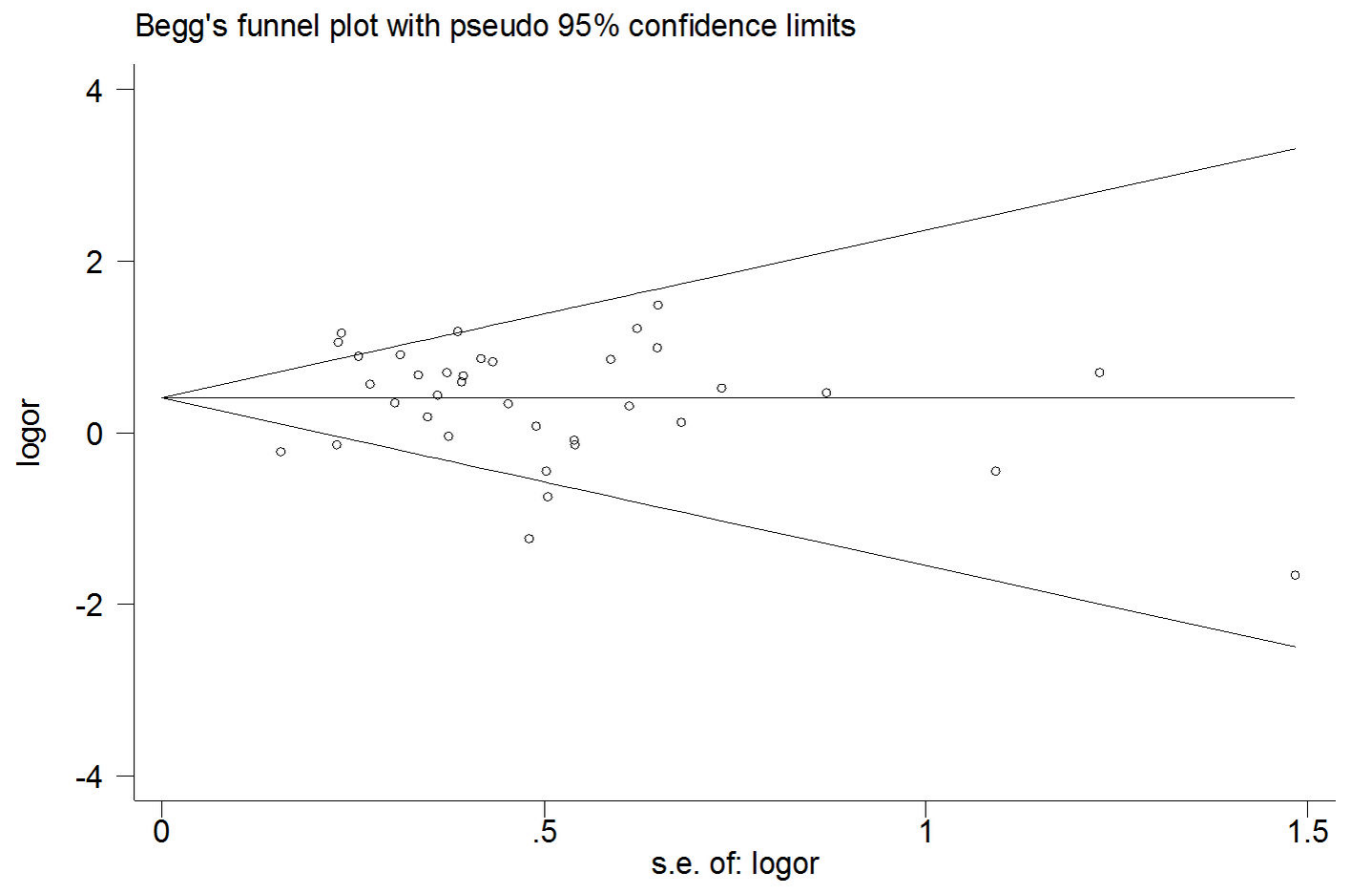
Begg’s funnel plot of CYP2C19 and cancer risk.

A single study involved in the meta-analysis was deleted each time to reflect the influence of the individual dataset to the pooled ORs, and the corresponding pooled ORs were not qualitatively altered (data not shown). The shape of the funnel plot did not indicate any evidence of obvious asymmetry (Figure 3), thus suggesting no publication bias among the studies included. Egger’s test was used to provide further statistical evidence; similarly, the results showed no significant publication bias in this meta-analysis (Egger test, t= 0.08, P = 0.93).

## Discussion

Cancer is a major public health problem in the world. Despite much investigation, detailed pathogenesis mechanisms of cancer remain a matter of speculation. Large sample and unbiased epidemiological studies of predisposition genes polymorphisms could provide insight into the in vivo relationship between candidate genes and complex diseases. This is the first comprehensive meta-analysis that examined the CYP2C19 polymorphisms and the relationship to cancer susceptibility. Its strength was based on the accumulation of published data giving greater information to detect signiﬁcant differences. In total, the meta-analysis involved 30 studies for cancer, which provided 11,554 cases and 16,592 controls. Our results indicated that the PMs genotypes of CYP2C19 is a risk factor for developing cancer.

In the stratiﬁed analysis by ethnicity, signiﬁcant associations were found in Asians, while no associations were found in Caucasians. Several factors may contribute to the results that the same polymorphism plays different roles in cancer risk among different ethnic populations. Above all, ethnic differences may attribute to these different results, since the distributions of the CYP2C19 polymorphism were different between various ethnic populations. For instance, the frequencies of CYP2C19*3 polymorphism allele differs from less than 0.5% in Caucasian population [36], 12% in Chinese population [22], to 16% in Japanese populations [17]. However, several studies conducted among Middle East populations which shown a similar PMs genotypes prevalence as Caucasians found a significant association between CYP2C19 PMs genotype and cancer susceptibility [21,27]. It is possible that variation at this locus has modest effects on cancer, but environmental factors may predominate in the progress of cancer, and mask the effects of this variation. On the other hand, study design or small sample size or some environmental factors may affect the results. Most of these included studies did not consider most of the important environmental factors. Thus, the effect of single genetic factor on the risk of cancer may be more pronounced in the presence of other common genetic or environmental risk factors such as smoking, hepatitis virus infection, *H. pylori* infection. Another explanation of no association between CYP2C19 polymorphism and cancer risk in Caucasians may be that different linkage disequilibrium patterns usually exist in different populations.

In another subgroup analysis by cancer types, we found that CYP2C19 PMs genotypes led to an increased incidence of esophagus cancer, gastric cancer, lung cancer and head neck cancer as well as hepatocellular carcinoma, but not for breast cancer, colorectal cancer, leukemia, prostate cancer, bladder cancer and biliary tract cancer. However, in our meta-analysis, only one or two studies were available for some speciﬁc cancers, and they had limited sample size, and hence the results may be capricious and should be interpreted with caution. It should also be considered that the apparent inconsistency of these results may underlie differences in ethnicity, lifestyle and disease prevalence as well as possible limitations due to the relatively small sample size. The current knowledge of carcinogenesis indicates a multi-factorial and multi-step process that involves various genetic alterations and several biological pathways. Thus, it is unlikely that risk factors of cancer work in isolation from each other. And the same polymorphism may play different roles in cancer susceptibility, because cancer is a complicated multi-genetic disease, and different genetic backgrounds may contribute to the discrepancy. And even more importantly, the low penetrance genetic effects of single polymorphism may largely depend on interaction with other polymorphisms and/or a particular environmental exposure.

After stratification by sample size, the association became non-signiﬁcant when the meta-analysis was restricted to larger studies (at least 500 cancer cases), suggesting a potential small study effects with an overestimate of the true association by smaller studies. Even though the use of a statistical test did not show publication bias among included studies, both theoretical arguments and empirical studies (including surveys and simulations) have demonstrated that the Egger’s test is not powerful enough to be used in publication bias assessment. Therefore, additional studies with much larger sample size are warranted to further validate our results.

When stratiﬁed by the source of controls, our results indicated a signiﬁcantly increased risk among studies using hospital-based controls but not for population-based controls. The reason may be that the hospital-based studies have some biases because such controls may just represent a sample of ill-deﬁned reference population, and may not be representative of the general population very well, particularly when the genotypes under investigation were associated with the disease conditions that the hospital-based controls may have. Therefore, using a proper and representative population-based control subjects is very important to reduce biases in such genetic association studies.

Though polymorphism in CYP2C19 largely accounts for the poor metabolizing status, it has also been reported to influence the metabolism, particularly detoxiﬁcation of the carcinogens [37]. Using hepatic microsomal preparations, CYP2C19 was shown to metabolize both aromatic amines (AA, nitrosamines) and polycyclic aromatic hydrocarbons (PAHs), found in tobacco smoke and smokeless tobacco [38–40]. Therefore, CYP2C19 polymorphism is considered as one of the factors that determine an individual’s cancer susceptibility by the interindividually different ability of detoxiﬁcation of carcinogen(s) and/or activation of procarcinogen(s) [41,42]. Homozygous EMs may have higher carcinogen level and potent cell toxicity by the higher ability for bioactivating procarcinogens, whereas PMs may have a higher carcinogen level and potent cell toxicity by the lower ability for detoxiﬁcating carcinogens. As for gastric cancer, most patients are infected with *H. pylori* and have severe active gastritis or atrophic gastritis, suggesting that candidate carcinogens metabolized by CYP2C19 require severe inﬂammation or atrophic changes induced by *H. pylori* infection in order to initiate cancerous transformation in gastric epithelial cells [18]. In animal models, chemical carcinogen-induced gastric cancer development was enhanced in the presence of atrophic gastritis caused by chronic *H. pylori* infection [43,44]. Therefore, it was assumed that the direct effect of candidate carcinogen(s) metabolized by CYP2C19 on the gastric epithelial cells is enhanced in the presence of *H. pylori* infection [18]. Recently, Wu et al. found that CYP2C19 mRNA expression is highest in hepatocarcinoma tissue, moderate in adjacent normal liver tissue [45]. The significantly elevated expression of CYP2C19 mRNA in hepatocarcinoma suggests an association between the occurrence of hepatocarcinoma and the expression and/or turnover of CYP2C19 mRNA [44].

As like any other meta-analysis, limitations also inevitably existed in the present study. Firstly, our results were based on unadjusted estimates, while a more precise analysis should be conducted if all individual raw data were available, which would allow for the adjustment by other co-variants including age, sex, drinking status, cigarette consumption and other lifestyle. Secondly, the subgroup meta-analyses considering different type of cancer and CYP2C19 were performed on the basis of a fraction of all the possible data to be pooled, so selection bias may have occurred and our results may be over inflated. Since studies among some specific types of cancer are currently limited, further studies including a wider spectrum of subjects should be carried out to investigate the role of the enzyme in different types of cancer. Thirdly, lacking the original data for the included studies limited our further evaluation of potential interactions among gene–gene, gene–environment, or even different polymorphism loci of the same gene, which all may affect cancer risk. Fourthly, hampered by limited number of studies available currently prevent us from making further analysis to identify any interactions between genetic variation and cancer risk as well as ethnic diversity. Finally, some genetic polymorphisms were not in HWE in the current meta-analysis, which may affect the validity of conclusion. These considerations may distort our results.

In summary, our meta-analysis demonstrated an association between CYP2C19 polymorphism and cancer risk among Asian populations, but not among Caucasians. As a statistically signiﬁcant 1.52-fold increased risk for cancer appeared for individuals with PM genotypes, this result suggests that in the presence of both of the two risk factors, an important number of cancer cases would occur. For future association studies, strict selection of patients, much larger sample size will be required. More studies should also be carried out to examine the impact of CYP2C19 on cancer risk, especially in Caucasian populations. Moreover, gene–gene and gene–environment interactions should also be considered in future studies.

## Supporting Information

Table S1 **Summary crude odds ratios (ORs) and 95% Confidence Intervals (95% CI) after applying the BFDP.**
(DOCX)

Checklist S1 (DOC)
