# Supplementary material for: Poor Metabolizers at the Cytochrome P450 2C19 Loci Is at Increased Risk of Developing Cancer in Asian Populations
Source: PLoS One. 2013 Aug 27;8(8):e73126. doi: 10.1371/journal.pone.0073126 (PMC3754911; doi:10.1371/journal.pone.0073126)
Supplement: Table S1 — Summary crude odds ratios (ORs) and 95% Confidence Intervals (95% CI) after applying the BFDP. (DOCX) [file pone.0073126.s001.docx]

**Table S1** Summary crude odds ratios (ORs) and 95% Confidence Intervals (95% CI) after applying the BFDP.

| ***OR (95% CI)*** | ***Observed***  ***p-value*** | ***Prior probabilities*** | | | | | |
| --- | --- | --- | --- | --- | --- | --- | --- |
|  |  | 0.05 | 0.01 | 10^-3^ | 10^-4^ | 10^-5^ | 10^-6^ |
|  |  | **Bayesian False Discovery Probability** | | | | | |
| 1.52 (1.23-1.88) | P<10^-4^ | 0.067 | 0.271 | 0.789 | 0.974 | 0.997 | 1.00 |
